# Multiple cause-of-death data among people with AIDS in Italy: a nationwide cross-sectional study

**DOI:** 10.1186/s12963-017-0135-3

**Published:** 2017-05-18

**Authors:** Enrico Grande, Antonella Zucchetto, Barbara Suligoi, Francesco Grippo, Marilena Pappagallo, Saverio Virdone, Laura Camoni, Martina Taborelli, Vincenza Regine, Diego Serraino, Luisa Frova

**Affiliations:** 1 Servizio Sistema integrato salute, assistenza, previdenza e giustizia, Istituto Nazionale di Statistica, Viale Liegi 13, 00198 Rome, Italy; 2 Unit of Cancer Epidemiology, CRO Aviano National Cancer Institute, Via Gallini 2, 33081 Aviano, PN Italy; 3 Centro Operativo AIDS, Istituto Superiore di Sanità, Viale Regina Elena 299, 00161 Rome, Italy

**Keywords:** HIV/AIDS, Cause-specific mortality, Multiple causes of death, Italy

## Abstract

**Background:**

Multiple cause-of-death (MCOD) data allow analyzing the contribution to mortality of conditions reported on the death certificate that are not selected as the underlying cause of death. Using MCOD data, this study aimed to fully describe the cause-specific mortality of people with AIDS (PWA) compared to people without AIDS.

**Methods:**

We conducted a nationwide investigation based on death certificates of 2,515 Italian PWA and 123,224 people without AIDS who had died between 2006 and 2010. The conditions most frequently associated with PWA mortality, compared to people without AIDS, were identified using an age-standardized proportion ratio (ASPR) calculated as the ratio between the age-standardized proportion of a specific cause among PWA and the same proportion among people without AIDS.

**Results:**

The most frequently reported conditions at death among PWA were infectious/parasitic diseases (52%), digestive (36%), respiratory (33%), and circulatory (32%) system diseases, and neoplasms (29%). All AIDS-defining conditions resulted highly associated (ASPR significantly greater than unity) with PWA deaths. Significant associations also emerged for leishmaniasis (ASPR = 188.0), encephalitis/myelitis/encephalomyelitis (ASPR = 14.3), dementia (ASPR = 13.1), chronic viral hepatitis (ASPR = 13.1), liver fibrosis/cirrhosis (ASPR = 4.4), pneumonia (ASPR = 4.4), anal (ASPR = 12.1) and liver (ASPR = 1.9) cancers, and Hodgkin’s disease (ASPR = 3.1).

**Conclusions:**

Study findings identified the contribution of several non-AIDS-defining conditions on PWA mortality, emphasizing the need of preventive public health interventions targeting this population.

## Background

In Italy, about 4,000 new HIV diagnoses and approximately 800 AIDS cases are reported yearly to the National HIV and AIDS surveillance systems, with a steady trend observed in the last decade. However, the distribution of new AIDS cases by transmission mode has changed over time, with a decreasing proportion of injecting drug users (31.2% in 2005 and 11.3% in 2015) and an increasing proportion of cases acquired through sexual contact (61.3% in 2005 and 79.8% in 2015) [1]. A total of 123,000 (115,000–145,000) individuals aged 15 years or more were estimated to be living with HIV in Italy at the end of 2012 with a prevalence of 0.28 (0.24–0.32) per 100,000 residents [2]. In the same year, 94,146 people diagnosed with HIV and referred to the national healthcare system were reported. The majority were males (70.1%), Italians (84.4%), and aged between 25 and 49 years (63.4%); the probable route of transmission was heterosexual contact in 37.5% of cases, injecting drug use in 28.1%, and male-to-male contact in 27.9%; 87.6% underwent highly active antiretroviral therapy (HAART) [3]. HAART introduction in the mid-1990s has led to marked improvements in survival of HIV-infected people [4] with consequent changes in mortality patterns. In particular, a growing proportion of deaths due to non-HIV/AIDS-related causes [5–8] has emerged over time.

The analysis of mortality by cause mainly refers to the underlying cause of death (UC), defined as the disease or injury that initiated the train of morbid events leading directly to death, or the circumstance of the accident or violence that produced the fatal injury [9]. However, the UC approach is often insufficient to properly analyze mortality, as it captures only the “tip of the iceberg.” In fact, it could divert the attention from conditions that tend to be reported as contributory causes. As a consequence, the contribution of these conditions to overall mortality could be underestimated [10–12].

On the other hand, the use of multiple cause-of-death (MCOD) data – i.e., all the diseases that the certifying physician has considered relevant to death – allow analyzing all the medical circumstances surrounding the death and enable a more exhaustive description of the cause-specific mortality patterns [13]. This is important for the whole population, but especially for specific subgroups characterized by elevated co-morbidity, such as people with AIDS (PWA). Despite the increasing availability of MCOD data in many countries and the growing number of studies focusing on MCOD, only few have taken into consideration people with HIV infection or AIDS [6, 14, 15].

This study aimed to comprehensively describe the mortality patterns of Italian PWA using MCOD data. Furthermore, it aimed to identify the specific conditions that are most frequently associated with PWA mortality, as compared to people without AIDS.

## Methods

This population-based, cross-sectional investigation compared the death certificates of PWA with those of people without AIDS, for the years 2006 to 2010. The two groups of death certificates were identified by linking two nationwide data sources: the Italian National AIDS Registry (RAIDS) and the National Register of Causes of Death (RCOD).

### Sources of data

The RCOD, managed by the Italian National Institute of Statistics (ISTAT), collects all death certificates issued in Italy. Individual death certificates consist of two sections: a medical section for certification of causes of death reported by a physician and a sociodemographic section filled in by local civil registration officers. Sociodemographic information refers to sex, age, place (municipality or foreign state) of birth, residence and place of death, citizenship, and education level. The cause-of-death certification includes the description of the sequence of conditions that directly lead to death and, where appropriate, other conditions that may have contributed but were not directly linked to death. The classification of causes of death and the selection of the UC is performed according to the rules and provisions included in the International Classification of Diseases, 10^th^ Revision (ICD-10) provided and updated by the World Health Organization (WHO). In recent years, ISTAT also disseminates MCOD data.

The RAIDS, managed by the National Institute of Health (ISS), collects information on PWA in Italy, according to the European AIDS case definition [16, 17]. The reporting of AIDS cases became mandatory in 1986. The RAIDS includes demographic information on date of AIDS diagnosis, sex, age at diagnosis, place of birth and residence at diagnosis; conversely, date of death is not mandatorily updated. The RAIDS has a national coverage; a previous study estimated a 6% underreporting by linking RAIDS data with the National mortality database [18].

### Record-linkage and definition of the study groups

In order to identify the death certificates referring to PWA, the RAIDS, updated to 31^st^ December 2010, was linked to the RCOD database for the period 2006–2010. The record-linkage was carried out in compliance with the national law regulating the use of data from PWA and according to the authorization of the Italian Data Protection Authority (provided in the National Statistical Plan).

The record-linkage was conducted using a semi-automated and validated procedure with an updated version of SALI software [19]. This is a deterministic matching procedure that relies on an automated multiple-step algorithm using blinded names, surnames, and date of birth as matching criteria. To optimize linkage sensitivity, SALI takes into account of most spelling errors; thus, subsequent manual comparison of other variables present in both databases (e.g., place of birth) is required to discard false-matched records. For the aims of the present study, two groups of deaths were identified through the record-linkage:

1. All the RCOD deaths matched to the RAIDS (3,681), i.e., the deaths of PWA, representing the study group (AIDS deaths);
2. All the RCOD deaths non-matched to the RAIDS (2,882,337), i.e., deaths of people without AIDS, further excluding deaths certificates with any mention of AIDS/HIV (ICD-10 codes B20-B24 and R75), representing the comparison group (non-AIDS/HIV deaths).

Furthermore, the following population subgroups were excluded from the analysis:

- Foreign citizens (295 death records among AIDS deaths, 26,623 death records among non-AIDS/HIV deaths), because of the linkage procedure lower sensitivity towards frequent spelling errors in foreign names/surnames and because of foreign citizens’ higher propensity to migrate (which causes frequent losses to follow-up);
- Death certificates from Trento and Bolzano provinces (7 records among AIDS deaths, 42,412 records among non-AIDS/HIV deaths), for which MCOD were not available.

The present MCOD analysis focused on deaths occurred in the 35–54-year age class, where AIDS mortality rates were the highest, and which included 78% of total deaths among PWA in the period 2006–2010. The number of death certificates excluded from the comparison group due to the mention of B20-B24 or R75 codes was 1,905.

In the end, the study population included 2,515 records of AIDS deaths (among these, 486 death certificates had no mention of AIDS/HIV) and 123,224 records of non-AIDS/HIV deaths.

### Statistical analysis

The conditions most frequently associated with PWA mortality were identified using an age-standardized relative risk indicator, which identifies the excess proportion of having a specific disease mentioned in the death certificate among PWA, as compared to those without AIDS/HIV.

For a specific cause of death *c,* the Age-Standardized Proportion Ratio (ASPR) was calculated as the ratio between the following estimated proportions:

1. ${\widehat{p}}_{Ac}$, proportion of death certificates mentioning the cause *c* among AIDS deaths *(A);*
2. ${\hat{p}}_{\overline{A} c}$, proportion of death certificates mentioning the cause *c* among non-AIDS/HIV deaths (*Ā*).

$$ ASP{R}_c=\frac{{\widehat{p}}_{A c}}{{\widehat{p}}_{\overline{A} c}} $$

The two estimated proportions were obtained through age standardization based on the 5-year age class distribution of total deaths aged 35–54 years, according to the following formulas:

$$ {\hat{p}}_{A c}={\displaystyle {\sum}_x\frac{{}_x{D}_{A c}}{{}_x{D}_A}\times {}_x w} $$

$$ {\hat{p}}_{\overline{A} c}={\displaystyle {\sum}_x\frac{{}_x{D}_{\overline{A} c}}{{}_x{D}_{\overline{A}}}\times {}_x w} $$

where:

*D*_*Ac*_ observed number of deaths certificates mentioning the cause *c* among AIDS deaths

*D*_*A*_ observed number of AIDS deaths

*D*_*Āc*_ observed number of deaths certificates mentioning the cause *c* among non-AIDS/HIV deaths

*D*_*Ā*_ observed number of non-AIDS/HIV deaths*.*

_*x*_ *w* proportion of deaths at age *x* (_*x*_ *D*) on the total number of deaths (*D*).

The 95% confidence intervals (CI) for the ASPR were calculated using the standard error formula for relative risks according to Altman [20]. ASPR significantly greater than unity indicates a higher frequency of a specific cause among AIDS deaths; conversely, values lower than unity indicate a higher frequency of the specific cause among non-AIDS/HIV deaths. Causes of death were analysed at a high level of specificity (ICD-10 category). Furthermore, the ASPR was calculated also for broad groups of causes (ICD-10 chapters).

## Results

**Fig. 1:**
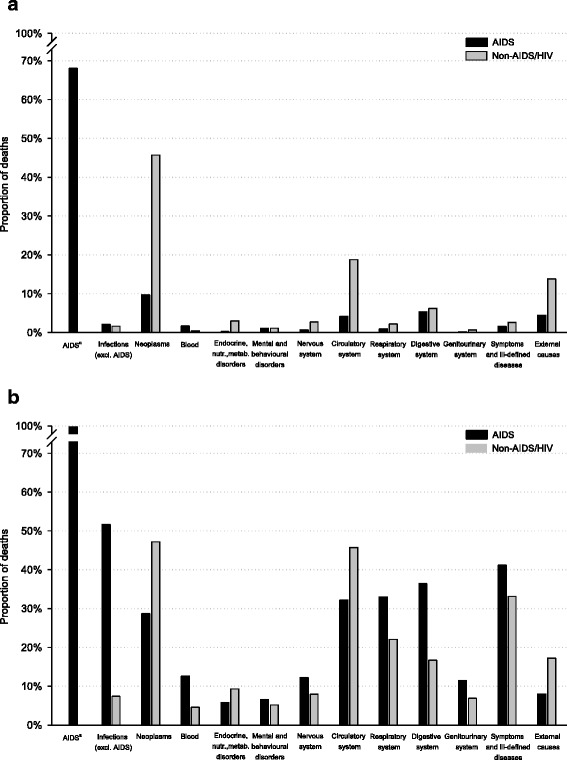
Underlying cause (**a**) and multiple cause (**b**)of death distribution: a comparison between AIDS deaths and non-AIDS/HIV deaths. Italy, period 2006–2010. **a** ICD-10 codes B20-B24. In the representation of multiple causes for AIDS deaths, all the 2,515 certificates matched to the RAIDS are assumed to mention a B20-B24 code

A comparison of UC and MCOD distribution between AIDS and non-AIDS/HIV deaths is presented in Fig. 1. The analysis of proportion of deaths by UC (Fig. 1a) provided evidence that AIDS deaths were mainly attributable to infectious diseases (68% to AIDS, 2% to other infectious diseases), while low proportions of deaths were due to neoplasms (10%) and diseases of the circulatory system (4%). This pattern strongly diverged from the one observed among non-AIDS/HIV deaths. Considering MCOD data (Fig. 1b), the more frequently mentioned causes in the death certificates of PWA were infectious diseases other than AIDS (52%), symptoms-signs and ill-defined diseases (41%), diseases of digestive (36%), respiratory (33%), and circulatory (32%) systems, and neoplasms (29%). The most frequently reported conditions in the comparison group were neoplasms and diseases of the circulatory system, mentioned in 47% and 46% of the certificates, respectively.

Table 1 shows crude proportions of certificates mentioning specific causes among AIDS and non-AIDS/HIV deaths and statistically significant values of ASPR with 95% CI.

**Table 1:** Multiple cause of death analysis, comparing certificates of AIDS deaths and non-AIDS/HIV deaths: crude proportions of deaths and age-standardized proportion ratio (ASPR)^§^, with corresponding 95% confidence intervals (CI), by any mentioned cause of death

Cause of death mentioned in the death certificate		Crude proportion (%)		ASPR	95% CI
ICD-10 code	Description	AIDS deaths (Total no. 2,515)	Non-AIDS/HIV deaths (Total no. 123,224)		
*A00-B99*	*Infectious and parasitic diseases (excluding B20-B24)* ^a^	*51.7*	*7.4*	*7.0*	*(6.7* – *7.3)*
B58	Toxoplasmosis	3.3	<0.1	4167.4	(583.4 – 29,769.1)
B45	Criptococcosis	1.8	<0.1	480.4	(192.1 – 1,201.2)
A31	Infection due to other mycobacteria	2.1	<0.1	330.3	(157.4 – 693.0)
B59	Pneumocystosis	3.1	<0.1	208.1	(129.6 – 334.2)
B55	Leishmaniasis	0.4	<0.1	188.0	(39.5 – 894.1)
A81	Atypical virus infections of central nervous system	2.7	<0.1	63.6	(44.4 – 91.1)
B25	Cytomegaloviral disease	4.8	0.1	62.9	(48.7 – 81.4)
B37	Candidiasis	4.0	0.1	50.1	(37.7 – 66.5)
A16	Respiratory tuberculosis. not confirmed bacteriologically or histologically	1.2	0.1	15.6	(10.5 – 23.1)
B18	Chronic viral hepatitis	23.9	1.8	13.1	(12.1 – 14.2)
A49	Bacterial infection of unspecified site	0.5	0.1	8.2	(4.3 – 15.8)
B44	Aspergillosis	0.4	0.1	6.5	(3.7 – 11.6)
B16	Acute hepatitis B	1.4	0.2	5.9	(4.1 – 8.5)
A41	Other septicemia	12.2	4.7	2.6	(2.4 – 2.9)
*C00-D48*	*Neoplasms* ^a^	*28.7*	*47.2*	*0.6*	*(0.6 – 0.7)*
C46	Kaposi’s sarcoma	2.7	<0.1	298.9	(162.4 – 550.1)
C83	Diffuse non-Hodgkin’s lymphoma	2.0	0.1	14.1	(10.2 – 19.4)
C21	Malignant neoplasm of anus and anal canal	1.0	0.1	12.1	(7.7 – 18.8)
C85	Other and unspecified types of non-Hodgkin’s lymphoma	8.4	1.3	6.5	(5.7 – 7.5)
C81	Hodgkin’s disease	1.0	0.3	3.1	(2.1 – 4.5)
C53	Malignant neoplasm of cervix uteri^b^	3.9	1.1	2.8	(1.7 – 4.6)
C22	Malignant neoplasm of liver and intrahepatic bile ducts	3.3	2.0	1.9	(1.6 – 2.3)
C34	Malignant neoplasm of bronchus and lung	2.9	7.9	0.4	(0.3 – 0.5)
C25	Malignant neoplasm of pancreas	0.4	2.3	0.2	(0.1 – 0.4)
*D50-D89*	*Diseases of the blood. immunological disorders*s^a^	*12.6*	*4.5*	*2.8*	*(2.5 – 3.1)*
D61	Other aplastic anemias	2.5	0.5	5.1	(4.0 – 6.5)
D70	Agranulocytosis	0.6	0.2	2.7	(1.6 – 4.6)
D69	Purpura and other hemorrhagic conditions	1.1	0.6	2.0	(1.4 – 2.8)
*E00-E90*	*Endocrine. nutritional metabolic diseases* ^a^	*2.5*	*4.8*	*0.6*	*(0.4 – 0.7)*
E14	Unspecified diabetes mellitus	1.7	3.8	0.5	(0.4 – 0.7)
*F01-F99*	*Mental and behavioral disorders* ^a^	*6.5*	*5.2*	*1.2*	*(1.0 – 1.4)*
F03	Unspecified dementia	1.0	0.1	13.1	(8.3 – 20.5)
F11	Mental and behavioral disorders due to use of opioids	0.7	0.2	4.2	(2.5 – 7.0)
F19	Mental and behavioral disorders due to multiple drug use and use of other psychoactive substances	2.7	0.7	3.4	(2.6 – 4.4)
F09	Unspecified organic or symptomatic mental disorder	0.4	0.2	2.0	(1.1 – 3.9)
F10	Mental and behavioral disorders due to use of alcohol	1.1	1.3	0.6	(0.4 – 1.0)
F32	Depressive episode	0.4	1.0	0.5	(0.3 – 0.8)
G00-H95	*Diseases of the nervous system and the sense organs* ^a^	*12.2*	*7.9*	*1.5*	*(1.4 – 1.7)*
G04	Encephalitis. myelitis and encephalomyelitis	2.3	0.2	14.3	(10.6 – 19.1)
G93	Other disorders of brain	6.4	2.8	2.4	(2.1 – 2.8)
G31	Other degenerative diseases of nervous system. not elsewhere classified	0.4	0.2	2.4	(1.4 – 4.2)
G62	Other polyneuropathies	0.4	0.2	2.2	(1.1 – 4.4)
*I00-I99*	*Diseases of the circulatory system* ^a^	*32.2*	*45.7*	*0.7*	*(0.7 – 0.7)*
I33	Acute and subacute endocarditis	0.4	0.1	2.8	(1.5 – 5.4)
I27	Other pulmonary heart diseases	1.2	0.4	2.5	(1.7 – 3.7)
I51	Complications and ill-defined descriptions of heart disease	4.1	5.4	0.7	(0.6 – 0.9)
I50	Heart failure	2.9	3.9	0.7	(0.6 – 0.9)
I61	Intracerebral hemorrhage	1.8	2.9	0.7	(0.5 – 0.9)
I 25	Chronic ischemic heart disease	1.9	4.7	0.5	(0.4 – 0.6)
I26	Pulmonary embolism	0.7	1.5	0.4	(0.3 – 0.7)
I64	Stroke. not specified as hemorrhage or infarction	0.4	1.1	0.3	(0.2 – 0.7)
I21	Acute myocardial infarction	2.1	7.5	0.3	(0.2 – 0.4)
I 49	Other cardiac arrhythmias	0.4	1.9	0.3	(0.2 – 0.5)
I10	Essential (primary) hypertension	1.1	4.9	0.3	(0.2 – 0.4)
*J00-J99*	*Diseases of the respiratory system* ^a^	*33.0*	*22.0*	*1.5*	*(1.5 – 1.6)*
J84	Other interstitial pulmonary diseases	2.7	0.5	6.2	(4.9 – 7.9)
J93	Pneumothorax	0.8	0.1	6.1	(3.9 – 9.6)
J15	Bacterial pneumonia. not elsewhere classified	0.5	0.1	6.0	(3.3 – 11)
J18	Pneumonia. organism unspecified	14.2	3.3	4.4	(4.0 – 4.9)
J80	Adult respiratory distress syndrome	1.0	0.4	2.5	(1.7 – 3.8)
J69	Pneumonitis due to solids and liquids	0.5	0.3	2.0	(1.2 – 3.4)
J44	Other chronic obstructive pulmonary disease	1.4	1.2	1.5	(1.1 – 2.0)
J96	Respiratory failure. not elsewhere classified	14.0	12.3	1.2	(1.0 – 1.3)
J81	Pulmonary edema	3.4	4.6	0.7	(0.6 – 0.9)
*K00-K92*	*Diseases of the digestive system* ^a^	*36.4*	*16.7*	*2.2*	*(2.1 – 2.3)*
K73	Chronic hepatitis. not elsewhere classified	2.5	0.2	11.0	(8.2 – 14.7)
K52	Other non-infective gastroenteritis and colitis	0.8	0.1	6.4	(4.1 – 9.8)
K74	Fibrosis and cirrhosis of liver	24.3	5.6	4.4	(4.1 – 4.7)
K85	Acute pancreatitis	1.0	0.4	2.5	(1.7 – 3.8)
K76	Other diseases of liver	6.5	3.0	2.2	(1.9 – 2.6)
K72	Hepatic failure. not elsewhere classified	11.3	5.6	1.9	(1.7 – 2.1)
K70	Alcoholic liver disease	1.0	1.8	0.6	(0.4 – 0.9)
K56	Paralytic ileus and intestinal obstruction without hernia	0.8	1.4	0.6	(0.4 – 0.9)
*N00-N99*	*Diseases of the genitourinary system* ^a^	*11.5*	*6.9*	*1.7*	*(1.5 – 1.9)*
N17	Acute renal failure	3.7	1.7	2.4	(2.0 – 2.9)
N19	Unspecified renal failure	4.9	3.3	1.5	(1.3 – 1.8)
*R00-R99*	*Symptoms signs and ill-defined causes (excluding R75)* ^a^	*41.2*	*33.1*	*1.2*	*(1.2 – 1.3)*
R16	Hepatomegaly and splenomegaly. not elsewhere classified	0.4	0.1	5.1	(3.0 – 9.0)
R64	Cachexia	16.0	4.2	3.9	(3.6 – 4.3)
R91	Abnormal findings on diagnostic imaging of lung	0.4	0.2	2.3	(1.3 – 4.0)
R57	Shock. not elsewhere classified	8.6	10.1	0.8	(0.7 – 1.0)
R40	Somnolence. stupor and coma	3.2	4.5	0.7	(0.6 – 0.9)
*V00-Y99*	*External causes of death* ^a^	*8.0*	*17.2*	*0.4*	*(0.4 – 0.5)*
X42	Accidental poisoning by and exposure to narcotics and psychodysleptics [hallucinogens] not elsewhere classified	1.0	0.3	2.6	(1.7 – 4.0)
X59	Exposure to unspecified factor	0.5	0.8	0.5	(0.3 – 0.9)
V89	Motor- or non motor-vehicle accident type of vehicle unspecified	0.4	2.7	0.1	(0.1 – 0.3)

Deaths of people aged 35–54 years. Italy, 2006-2010

^§^Calculated as the age-standardized ratio between the proportion of deaths reporting a selected cause among AIDS deaths and the same proportion among non-AIDS/HIV deaths

^a^ICD-10 chapter. The values of the proportion for the specific ICD-10 categories do not sum up to the totals by ICD-10 chapter because 1) only causes of death with a statistically significant ASPR and with at least 10 cases among AIDS deaths are shown and 2) each death certificate could report more than one cause within the same ICD-10 chapter

^b^Only females

The analysis highlighted, as expected, high values of the ASPR for all the AIDS-defining conditions, which are rare among non-AIDS/HIV deaths. The condition most strongly associated with AIDS was toxoplasmosis (ASPR = 4,167; 95% CI: 583.4–29,769.1), in addition to other AIDS-defining infections such as criptococcosis (ASPR = 480.4; 95% CI: 192.1–1,201.2), other mycobacterial infections (other than tuberculosis and Hansen disease; ASPR = 330.3; 95% CI: 157.4–693) and pneumocystosis (ASPR = 208.1; 95% CI: 129.6–334.2). Besides infectious diseases, a very strong association was observed for Kaposi sarcoma (KS; ASPR = 298.9; 95% CI: 162.4–550.1) and –although with lower ASPR values compared to KS– diffuse and other/unspecified non-Hodgkin lymphoma (NHL; ASPR = 14.1 and 6.5, respectively), cachexia (ASPR = 3.9), and cervical cancer (ASPR = 2.8).

Among non AIDS-defining diseases, the infectious conditions with the highest ASPR were leishmaniasis (ASPR = 188; 95% CI: 39.5–894.1) and chronic viral hepatitis (ASPR = 13.1; 95% CI: 12.1–14.2). This latter condition was reported in 23.9% of AIDS deaths (600 cases; of these, 586 reported hepatitis C virus) versus 1.8% in the comparison group. High values of ASPR were also observed for chronic hepatitis not stated as viral (11.0; 95% CI: 8.2–14.7). ASPR was also high for encephalitis, myelitis and encephalomyelitis (14.3; 95% CI: 10.6–19.1) and unspecified dementia (13.1; 95% CI: 8.3–20.5).

Among non-AIDS defining cancers, only anal (ASPR = 12.1; 95% CI: 7.7–18.8) and liver (ASPR = 1.9; 95% CI: 1.6–2.3) showed values of ASPR significantly higher than unity. Regarding the entire group of neoplasms, instead, the analysis showed a highest frequency among non-AIDS/HIV deaths (ASPR = 0.6; 95% CI: 0.6–0.7), as for two major cancer sites, i.e. lung (ASPR = 0.4; 95% CI: 0.3–0.5) and pancreas (ASPR = 0.2; 95% CI: 0.1–0.4).

Other cancer sites very frequently reported in the 35-54-year age group of the general population but poorly mentioned in AIDS deaths (less than 10 cases, and therefore not shown in Table 1) were female breast (19.3% in non-AIDS/HIV women and 0.9% in AIDS ones) (ASPR = 0.03; 95% CI: 0.01–0.09), colon (ASPR = 0.1; 95% CI: 0.03–0.16), stomach (ASPR = 0.1; 95% CI: 0.1–0.2), and brain (ASPR = 0.04; 95% CI: 0.01–0.15) cancers.

Moreover, the analysis highlighted some conditions very frequently reported among AIDS deaths, but with relatively low values of ASPR, particularly fibrosis/cirrhosis and hepatic failure (mentioned in the 24.3% and 11.3% of certificates of AIDS deaths, respectively; ASPR = 4.4; 95% CI: 4.1–4.7 and ASPR = 1.9; 95% CI: 1.7–2.1, respectively) and pneumonia (proportion among AIDS deaths 14.2%; ASPR = 4.4, 95% CI: 4.1–4.9).

Also, mental and behavioral disorders due to psychoactive drugs and renal failure resulted significantly more frequent among AIDS deaths.

Concerning circulatory system diseases, only acute and subacute endocarditis (ASPR = 2.8; 95% CI: 1.5–5.4) and other pulmonary heart diseases (ASPR = 2.5; 95% CI: 1.7–3.7) were significantly associated.

Unspecified diabetes mellitus was less frequently mentioned in AIDS deaths than in non-AIDS/HIV deaths (ASPR = 0.5; 95% CI: 0.4–0.7).

Among AIDS deaths, a lower frequency of external causes was observed (ASPR = 0.4; 95% CI: 0.4–0.5), but a significantly higher association for accidental poisoning by narcotics and psychodysleptics [hallucinogens] emerged (ASPR = 2.6; 95% CI: 1.7–4.0). Suicide by hanging – frequently mentioned among non-AIDS/HIV (proportion = 2.4%) – showed a low value of the ASPR (0.2; 95% CI: 0.1–0.3, data not shown).

## Discussion

This is the first time that MCOD data have been used to investigate cause-specific mortality of PWA in Italy, providing a detailed picture of the causes listed in their death certificates. The analysis of MCOD, compared to the use of UC data only, stressed the leading role of infectious diseases among the causes of death for AIDS patients, and it also showed the major contribution to death of other causes (e.g., diseases of digestive, respiratory, nervous, and genitourinary systems) that were more frequently mentioned in AIDS than in non-AIDS/HIV death certificates. Moreover, although neoplasms and diseases of the circulatory system were not frequently the UC in PWA (10% and 4% of deaths, respectively), their contribution to death rose when MCOD were considered (respectively mentioned in 29% and 32% of AIDS deaths).

### Infectious and parasitic diseases

Our results showed that although infectious diseases provided a small contribute to the UC among PWA, they represented the most common cause of death when considering the MCOD (Fig. 1); this result stressed the relevant role of these conditions (which are, in most cases, preventable or treatable) in the survival of HIV-infected individuals. The high proportion of infectious diseases among AIDS deaths has to be attributed in part to the immunodeficiency caused by HIV infection [21] and in part to behaviors that expose HIV-positive individuals to certain types of infections, such as viral hepatitis [22, 23]. In Italy, the HIV epidemic has been driven mainly by injecting drug users until a decade ago; some behaviors associated with drug use, such as alcohol abuse or non-sterile drug paraphernalia sharing, may have contributed to an increased risk of liver damage or parenteral acquisition of viral hepatitis infections [6, 15, 24]. Actually, chronic viral hepatitis was reported in about one-fourth of all death certificates of PWA. Of relevance, hepatitis C virus (HCV) was the most frequent causative agent of chronic viral hepatitis deaths, underscoring the need of an early management of HIV-HCV co-infected individuals [25].

Extremely high values of ASPR were observed for conditions included in the AIDS-defining disease list [16, 17]: toxoplasmosis, criptococcosis, infections due to other mycobacteria (other than tuberculosis and Hansen disease), pneumocystosis, cytomegaloviral disease, candidiasis, and respiratory tuberculosis. However, the contribution of each of these conditions was quite small in terms of proportion among infectious MCOD.

### Neoplasms

Neoplasms, in particular AIDS-defining ones, are among the most common underlying causes of death among people with HIV infection or AIDS [6, 8]. The use of MCOD, as compared to UC, clearly highlighted that the frequency of cancer diagnoses reported in the certificates of AIDS deaths is noteworthy (i.e., 29%), and largely variable according to cancer sites/types. In addition to AIDS-defining cancers (i.e., KS, NHL, and cervical carcinoma), other cancers known to be frequently incident among HIV-infected people turned out to be frequently reported also in death certificates of PWA. The ASPR values for these cancers ranged from about 300-fold for KS to about 2- or 3-fold for cervical carcinoma, Hodgkin lymphoma, anal, and liver carcinomas. Conversely, statistically significant reduced ASPR emerged for very common cancers in the general population (e.g., breast cancer in women, prostate cancer in men, colon-rectal, pancreatic cancers, and lung cancer). Findings regarding lung cancer, in particular, were apparently in contrast with the literature, since people with HIV/AIDS consistently reported higher incidence of lung cancer in Italy and elsewhere [26, 27]. Furthermore, in a previous UC analysis among Italian PWA, a 6.6-fold elevated risk of death for non-AIDS defining cancers was documented as compared to the general population, including a 5.9-fold higher death risk for lung cancer [28]. Nevertheless, the MCOD analysis allowed to explore the frequency of non-AIDS defining cancers when they were not UC. These findings underlined that a direct comparison of our findings – obtained by means of MCOD approach – with those from previous studies based on the risk of dying for specific UC may be misleading [6, 8]. The results of our analysis seemed thus to stress the strong overall burden of cancer among the causes of death, pointing to the importance of cancer prevention in people with HIV infection and AIDS to enhance their life expectancy.

### Diseases of the blood and immunological disorders

Overall, 13% of death certificates of deceased PWA reported one or more diseases of the blood and/or immunological disorders – a proportion that was 3-fold higher than among deceased people without AIDS/HIV. Notably, anaplastic and hemorrhagic conditions turned out to be associated with highly positive ASPR, a potentially surrogate marker of HIV-immunosuppression. Although a comparison with similar investigations is made difficult, it seems that our findings are in agreement with the existing evidence indicating that anemia is a frequent complication of HIV infection associated with an elevated risk of death [29].

### Endocrine, nutritional metabolic diseases

A negative association between unspecified diabetes mellitus and AIDS was found, due to a double proportion of this disease among non-AIDS deaths compared to AIDS deaths. The proportion of diabetes mellitus reported among non-AIDS deaths was consistent with the results from the PASSI Italian Surveillance System [30], suggesting that this condition could be underreported among AIDS deaths because deemed not relevant to death. Despite the high association reported in the literature between diabetes and HAART therapy [31], the low ASPR found for diabetes among AIDS deaths could also be due to a to a large number of late presenters without therapy.

### Mental and behavioral disorders

The proportion of mental and behavioral disorders resulted similar between the two study groups, although a high value of ASPR for dementia was observed. Dementia is commonly associated to AIDS. It could be caused by specific characteristics of HIV that reaches the central nervous system, immediately after primary infection, and plays a central role in determining pathological alterations and neurocognitive deficits [32]. The prevalence of neurocognitive disorders associated to HIV remains high in the HIV population even in the HAART era, due primarily to the persistence, even among treated patient, of forms of HIV associated neurocognitive disorders (HAND) [33]. European data confirm the high prevalence of HAND in HIV treated population, which amounts to 69% of the HIV-positive population with suppression plasma viremia [34].

Mental and behavioral disorders due to psychoactive drugs also resulted highly associated with AIDS: historically, injecting drug users are indeed the first Italian group which acquired the HIV infection [17].

### Diseases of the nervous system

Among diseases of the nervous system, the highest value of the ASPR corresponded to encephalitis, myelitis and encephalomyelitis, indicating that these causes are about 14-fold more frequent in AIDS deaths than in non-AIDS/HIV deaths [35]. These conditions could be a consequence to many infectious AIDS related diseases such as herpes simplex virus, cytomegalovirus, and toxoplasmosis [16]. Furthermore, recent studies showed that HIV can cause direct damage to the brain [36].

### Diseases of the circulatory system

Diseases of the circulatory system were more frequently reported among non-AIDS/HIV deaths than among AIDS deaths. However, they were mentioned in about 32% of death certificates of PWA. This remarkable proportion is consistent with the role of circulatory system diseases (especially cardiovascular diseases) as causes of death in people with AIDS or HIV infection described in previous studies [5, 6].

The only two diseases with a higher frequency among AIDS deaths, i.e., acute and subacute endocarditis and other pulmonary heart diseases, are very common cardiac complications due to injecting drug use [37].

Values of ASPR for ischemic heart diseases and heart failure, in both cases lower than unity, are in agreement with the results obtained by Whiteside and colleagues (2015) in comparing mortality rates of people with HIV infection to those of the general population in the US, by means of MCOD data.

### Diseases of the respiratory system

With regard to the frequency of respiratory conditions, it is worth noting that one out of three AIDS deaths had a respiratory disease listed in the death certificate – a frequency magnitude concealed when the UC approach is used. Pneumonia (organism unspecified) wasthe most common listed respiratory condition, in agreement with what was observed by Schwarz (2014) in the same period.

### Diseases of the digestive system

Hepatic diseases accounted for the largest proportion of causes of death and showed significantly high proportions among AIDS deaths. Liver diseases are one of the most frequent non-AIDS-defining causes of death in people living with HIV for a number of reasons: the high prevalence of hepatotropic viral co-infections, the hepatotoxicity of antiretroviral drugs, and the emergence of new liver conditions, including non-alcoholic fatty liver disease and non-cirrhotic portal hypertension [38]. Considering the high association between HIV and hepatic viral infections [39], it can be hypothesized that death certificates reporting chronic viral hepatitis in the group of infectious diseases would also frequently report a liver-associated condition coded in the group of diseases of the digestive system. The contribution of concurrent liver-related causes of death would suggest that hepatic viral co-infections and chronic liver damage also constitute a relevant cause of death among Italian PWA.

### Diseases of the genitourinary system

Genitourinary diseases were rarely mentioned as UC, but they appeared to be listed in about 11% of death certificates of PWA when the MCOD approach was used. Our finding is in agreement with previous evidence showing that up to 3% of HIV-infected patients may suffer of chronic kidney diseases in the HAART era [40]. The MCOD approach seems, thus, to offer an important opportunity to quantify the prevalence of renal failure, in particular, and to stress the importance of screening for renal disorders as a clinical challenge in the management of people with HIV/AIDS. Furthermore, genitourinary diseases were more frequently mentioned in the death certificates of PWA than among those of non-AIDS/HIV people, in agreement with other studies showing that people with AIDS or HIV infection are at higher risk of death for renal diseases [6, 15].

### Symptoms, signs, and ill-defined causes

Symptoms, signs, and ill-defined causes were reported in about 40% of certificates for AIDS deaths: the condition most frequently mentioned was cachexia, typically linked to the wasting syndrome, as reported in other international studies on AIDS deaths [41].

### External causes

Among external causes of deaths, only “accidental poisoning by and exposure to narcotics psychodysleptics” showed positive association with AIDS due to overdose among injecting drug users, that constituted the majority of Italian AIDS cases reported from the beginning of the epidemic [17].

The main strengths of this study were the national coverage of the population-based registries used to identify people with and without AIDS, the AIDS case definition based on official diagnoses mandatorily reported to the RAIDS, and the use of MCOD data.

The exclusion from the analysis of death certificates from the Trento and Bolzano provinces is expected to not affect the results, since the number of cases from these areas is expected to be very small (overall UC deaths due to AIDS account for 0.8% of UC deaths due to AIDS in Italy in the period 2006–2010).

The choice to focus on deaths aged 35–54 years enabled feasible (robust) MCOD analysis by detailed cause-of-death category. Furthermore, it prevented possible bias due to misdiagnosis of HIV/AIDS in people at older ages, which could result in undiagnosed AIDS cases among the comparison group. However, future developments of the study will include the analysis of deaths of PWA aged 55 years and over, in order to investigate also the mortality pattern of older AIDS patients.

A quota of death certificates were not matched to the RAIDS mentioned codes B20-B24 or R75 (1,905) and were then excluded from the comparison group. These cases are currently under investigation to establish how many of them referred to AIDS deaths or, more plausibly, to deaths of people reported with HIV infection. This in-depth analysis requires to take into account both multiple cause codes and the text reported by the certifier.

Only a really small number of death certificates of PWA reported codes B20-B24 or R75 as single cause of death (8 out of 2,515), without any impact on the validity of MCOD analysis.

The ASPR compares proportions of deaths and needs only mortality data without the risk exposed population. The indicator highlights the conditions most frequently mentioned in AIDS versus non-AIDS/HIV deaths. Therefore, the ASPR does not measure the overall mortality risk of PWA compared to the general population, as in other analyses [15]. Briefly, it is a measure of the propensity of a condition to act as a cause of death in AIDS patients. In addition, it is useful in detecting the conditions reported in the chain of events leading to death.

It is well known that mortality data derived from death certificates have some limitations. Among these, the death certificate completion is entrusted to the medical knowledge of the certifying physician, and it can suffer from a lack of specificity and/or underreporting [42]. On the other hand, the death certificates are the most relevant tool for understanding which causes of death are the most prevalent in a given population, and this information is central to resource allocation for prevention and treatment.

## Conclusions

The present study is the first application of MCOD analysis to PWA in Italy. This approach, based on all the conditions that were directly responsible for the death or may have contributed to it, allowed identifying the circumstances leading PWA to death in a more comprehensive picture compared to the traditional UC analysis. From a public health perspective, interventions targeted at preventing all causes of death beside the UC can lead to both improvements in the health status and mortality reductions for a given population. Indeed, many of the contributing causes the MCOD approach allowed to focus on, are consequences or complications of the UC (or its therapy) to which tertiary prevention is typically addressed (for instance cardiovascular diseases as consequence of HAART therapy). Study findings could be of aid in planning public health interventions oriented to the prevention of death among PWA in the late HAART era, by directing the attention to the role of non-AIDS-defining conditions.

## Abbreviations

ASPR: Age-standardized proportion ratio
CI: Confidence Interval
HAART: Highly active antiretroviral therapy
HCV: Hepatitis C virus
ICD-10: International classification of diseases), 10^th^ revision
ISS: National Institute of Health
ISTAT: Italian National Institute of Statistics
KS: Kaposi sarcoma
MCOD: Multiple cause of death
NHL: Non-Hodgkin lymphoma
PWA: People with AIDS
RAIDS: Italian National AIDS Registry
RCOD: National register of causes of death
UC: Underlying cause of death
WHO: World Health Organization
